# Therapeutic Potential of MicroRNA: A New Target to Treat Intrahepatic Portal Hypertension?

**DOI:** 10.1155/2014/797898

**Published:** 2014-04-09

**Authors:** Can-Jie Guo, Qin Pan, Hua Xiong, Yu-Qi Qiao, Zhao-Lian Bian, Wei Zhong, Li Sheng, Hai Li, Lei Shen, Jing Hua, Xiong Ma

**Affiliations:** ^1^Division of Gastroenterology and Hepatology, School of Medicine, Shanghai Institute of Digestive Disease, Renji Hospital, Shanghai Jiaotong University, 145 Shandong Middle Road, Shanghai, 200001, China; ^2^Digestive Disease Laboratory, Department of Gastroenterology, School of Medicine, Xinhua Hospital, Shanghai Jiaotong University, Shanghai 200092, China

## Abstract

Intrahepatic portal hypertension accounts for most of the morbidity and mortality encountered in patients with liver cirrhosis, due to increased portal inflow and intrahepatic vascular resistance. Most treatments have focused only on portal inflow or vascular resistance. However, miRNA multitarget regulation therapy may potentially intervene in these two processes for therapeutic benefit in cirrhosis and portal hypertension. This review presents an overview of the most recent knowledge of and future possibilities for the use of miRNA therapy. The benefits of this therapeutic modality—which is poorly applied in the clinical setting—are still uncertain. Increasing the knowledge and current understanding of the roles of miRNAs in the development of intrahepatic portal hypertension and hepatic stellate cells (HSCs) functions, as well as their potential as novel drug targets, is critical.

## 1. Introduction

Portal hypertension is one of the more common and severe complications that develops in patients with chronic liver diseases. The most common intrahepatic cause is cirrhosis [1]. The subsequent increases in portal venous inflow and intrahepatic vascular resistance are major factors for the maintenance of portal hypertension [2]. The mechanisms underlying these processes are incompletely understood. However, hepatic stellate cells (HSCs) have been shown to be involved and are regulated by many signal transduction pathways and genes, including transforming growth factor-beta (TGF-*β*)/SMAD, platelet-derived growth factor (PDGF), and vascular endothelial growth factor (VEGF) [3–5].

Unfortunately, no nonsurgical treatments have been validated for these multiple pathways and targets simultaneously. However, miRNA therapy offers novel possibilities. miRNAs are small noncoding RNAs of 21–25 nt, which usually negatively modulate gene expression at the posttranscriptional level by incomplete or complete complementary binding to target sequences within the 3′ untranslated region (UTR) of miRNA [6]. More than 30% of all genes are estimated to be miRNA-regulated. In contrast to traditional agents that target one specific protein [7], miRNAs exhibit a unique multitargeted pattern of action. Complementarity between the “seed sequence” of a single miRNA and the 3′ UTR of multiple genes, most of which are recognized as members of signal pathways, results in the downregulation of mRNA and/or protein levels. Thus, miRNAs may serve as the simultaneous regulator of multiple genes and their subsequent signaling pathways.

Moreover, an accumulating body of evidence suggests that miRNAs are associated with a wide range of cellular processes, including angiogenesis, cell growth, cell proliferation, and vascular integrity, that have been extensively analyzed in hepatic cells or tissues. Divergent miRNA patterns were observed during chronic liver diseases of various etiologies [8, 9]. In this regard, we have reviewed the growing body of evidence that suggests miRNA involvement in the development of intrahepatic portal hypertension and biological behavior of HSCs.

## 2. HSCs and Intrahepatic Modulation of Portal Pressure

Portal hypertension, a major complication of cirrhosis, is caused by both augmented intrahepatic vascular resistance and increased portal blood flow [10]. Accumulating evidence from in vitro and in vivo studies suggests that HSCs are key players in the pathogenesis of increased intrahepatic vascular resistance and blood flow in chronic liver diseases, in which HSCs proliferate, acquire characteristics of contractile cells, and undergo transdifferentiation into a myofibroblast phenotype [11]. In the normal liver, HSCs are located in the perisinusoidal space (space of Disse) beneath the endothelial barrier. Because of this anatomical location, resting HSCs may also play a role in modulating intrahepatic vascular resistance and blood flow at the sinusoidal level, although with limited capacity to contract or relax in response to various vasoactive mediators [12]. After acute or chronic injury to the liver, HSCs are activated, and their morphological and physiological characteristics change dramatically during myofibroblastic transdifferentiation. The most important phenotypic alterations of activated HSCs, as defined by their actions in liver fibrogenesis, are the proliferation of autocrine and/or paracrine proinflammatory, profibrogenic, and promitogenic cytokines; inordinate extracellular matrix (ECM) synthesis and secretion; resistance to apoptosis; increased contractility; and others [13].

The initial event in the pathophysiology of portal hypertension is increased vascular resistance to portal blood flow, which is caused primarily by structural changes, such as fibrotic scar tissue and regenerative nodules compressing portal and central venules [10]. Furthermore, swelling of hepatocytes and capillarization of hepatic sinusoids (loss of endothelial fenestrations and collagen deposition in the space of Disse) also contribute to increased vascular resistance [11]. Although architectural changes are prominent, several studies have shown a variable, dynamically activated HSC contractility, as well as compression of the sinusoids and the space of Disse, which significantly contribute to the increased pressure within the sinus and increased intrahepatic resistance typical of portal hypertension [14]. The imbalance between endogenous vasoconstrictors (such as endothelin-1, angiotensin II, thrombin, *α*-adrenergic stimuli, and substance P) and vasodilators (including nitric oxide (NO), H_2_S, somatostatin, and carbon monoxide (CO)) is responsible for the dynamically activated HSC contractility [15].

**Figure 1:**
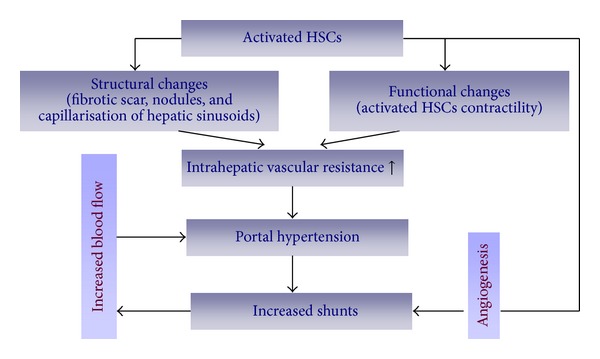
HSCs and intrahepatic modulation of portal pressure.

According to Ohm's law (Δ*P* = *Q* × *R*), the change in portal pressure along a vessel (Δ*P*) equals the product of the portal blood flow (*Q*) and the resistance to flow (*R*) [16]. In the normal liver, intrahepatic resistance changes with variations in portal blood flow, thereby keeping portal pressure within normal limits. In hepatic cirrhosis, however, intrahepatic resistance and splanchnic blood flow are both increased. Therefore, portal hypertension is caused by a combination of decreased compliance and increased portal blood flow. Although the hyperdynamic circulation in the splanchnic blood vessels is mainly responsible for the increased blood flow in the portal vein and contributes to its maintenance and aggravation in a more advanced stage of portal hypertension, the hepatic sinusoid, as principal site of blood flow regulation, is a potential target in intrahepatic modulation of portal pressure [17]. Due to the anatomical location of HSCs, which embrace the sinusoids and provide a favorable arrangement for sinusoidal constriction, HSCs play a critical role in modulating this increased blood flow at the sinusoidal level. Meanwhile, HSCs can also produce angiogenic molecules (such as VEGF and angiopoietin-1), thereby stimulating pathological sinusoidal remodeling and vascular structural changes [18]. Some investigators showed that small molecule inhibitors of receptor tyrosine kinases that target the growth factor pathways leading to angiogenesis and sinusoidal remodeling (i.e., VEGF, PDGF, and angiopoietin-1) are capable of lowering blood flow, most likely through a dual and converging antifibrogenic and antiangiogenic mechanism of action that affects HSCs [19] (Figure 1).

## 3. miRNA Regulation Linked to Biological Behavior of HSCs

During the process of hepatic injury, HSCs are known to be activated or “transdifferentiated” to myofibroblast-like cells, which play a pivotal role in ECM remodeling and hepatic blood flow regulation [20]. Recent studies have attempted to reveal the mechanism underlying HSC activation. As a result, hundreds of genes relevant to various functions have been reported to play a role in the process [21]. However, full understanding of HSC activation remains beyond our reach because of its complexity, especially considering the intricate regulation of gene expression. Fortunately, identification of multiple miRNAs, along with a comprehensive description of the miRNA/mRNA interaction network, may add to our knowledge of gene regulation throughout HSC activation. Microarray hybridization and quantitative RT-PCR analysis identified numerous miRNAs that are differentially expressed during HSC activation. Data are summarized from different studies that revealed 47 significantly upregulated (miR-874, 29C*, 501, 349, 325-5p, 328, 138, 143, 207, 872, 140, 193, Let-7a, let-7b, let-7c, let-7e, 125b, 130a, 130b, 132, 145, 152, 184, 199a, 199a-3p, 199a-5p, 21, 210, 214, 218, 22, 221, 222, 27a, 27b, 30a, 30c, 30d, 301a, 31, 34b, 34c, 345-5p, 349, 425, 450, and 455) and 53 significantly downregulated miRNAs (miR-341, 20b-3p, 15, 15b, 16, 375, 122, 146, 146a, 92a, 92b, 126, 126*, Let-7f, 10a, 10a-5p, 101a, 122a, 125a, 139-5p, 150, 151*, 181a, 187, 19a, 19b, 192, 194, 195, 207, 26a, 26b, 29a, 29b, 29c, 30a-5p, 30b, 30c, 30d, 301, 335, 355, 338, 378, 422b, 450a, 483, 497, 520b, 520c, 721, 877, and 9) in rat HSCs during activation [22–28]. miRNAs with and without asterisks are derived from the same precursor miRNA. Increasing evidence indicates that these miRNAs target genes that are implicated in a variety of biological processes, including cell proliferation, cell differentiation, cell cycle regulation, and apoptosis [29–31].

Previously, we have reported that miR-15/16 is involved in regulating apoptosis and proliferation in activated HSCs by interfering with the expression of Bcl-2 and CCND1, thereby mediating resistance to apoptosis and cell cycle arrest [26, 29]. Further, miR-150, -194, -146a, -29, -195, and -19b have demonstrated inhibitory effects on both fibrogenesis and proliferation of activated HSCs, while overexpression of miR-21, -27a/b, and -181b has been shown to result in cell proliferation [23, 30–37]. HSC activation is also characterized by accumulation of excess ECM components and fatty acids, which disrupt liver microcirculation and lead to liver injury [38, 39]. MiR-29b has been identified as the most effective suppressor of type I collagen (Col1A1) at the mRNA and protein level via its direct binding to the Col1A1 3′ UTR [40]. An increasing number of studies have proved that hepatic lipid metabolism irregulation increases hepatic endocannabinoid production, promotes hepatic fibrogenesis, enhances the hepatic vasoconstrictive response to endothelin-1, and aggravates hepatic microcirculatory dysfunction; these events subsequently increase intrahepatic resistance and portal hypertension in nonalcoholic steatohepatitis cirrhotic rats [41]. miRNAs have now been identified as potent posttranscriptional regulators of lipid metabolism genes involved in cholesterol homeostasis and fatty acid oxidation. For example, retinoid X receptor alpha, which is indicated to be a new regulator in fat metabolism and cell proliferation during HSC activation, was confirmed to be the target of miR-27a and -27b [30]. These findings may not only highlight the essence of portal hypertension due to HSC activation, but may also facilitate novel therapeutic strategies against portal hypertension and offer insight into its progression (Table 1).

Apart from the miRNA mimics, miR-128 inhibitor was employed to uncover the 157 transcripts downregulated by miRNA [44]. Gene silencing of dicer (a key enzyme for miRNA maturation) further highlights the global action of miRNA inhibition in HSCs. In detail, inhibition of dicer led to the significant reduction of miR-138, -143, -140, and -122 levels, of which miR-138 exhibited the strongest decline. Many fibrosis-related genes, including phosphatase and tension homolog deleted on chromosome 10 (PTEN), Ras GTPase activating-like protein 1 (RASAL1), acyl-CoA synthetase long-chain family member 1 (ACSL1), and p27, are regulated at the mRNA level after being targeted by differentially expressed miRNAs. Suppression of collagen synthesis in activated HSCs occurs as a result [45]. miRNA inhibitors, therefore, are indicated as another approach to the antifibrosis treatment.

## 4. Signaling Pathways and Key Factors Regulated in HSCs by miRNAs

Recent studies have attributed HSC activation to the regulation of many signal transduction pathways, including lipid metabolism and cell cycle regulation, and the signaling pathways of TGF-*β*/SMAD, PDGF, nuclear factor kappa-light-chain-enhancer of activated B cells (Nf-*κ*B), mitogen-activated protein kinase (MAPK), Wnt, VEGF, and others [46–50]. Furthermore, changes in miRNAs and their inhibitory effect on gene expression, especially those relevant to signal transduction, add to our knowledge of the regulatory mechanisms of HSC activation. Bioinformatic interpretation [51] revealed that 13 signal transduction pathways were overrepresented, while 22 were downregulated, in the activation of HSCs. Some of the signal transduction pathways have been shown to play a significant role in this process, of which TGF-*β* and PDGF/VEGF-like growth factor are likely the most important [52, 53]. It is widely accepted that early proliferative responses in HSC activation are mainly mediated by TGF-*β*/SMAD pathways. TGF-*β*-mediated HSC activation is, in general, considered to be the ECM producer responsible for fibrogenesis and has been identified to be a mechanistic factor in intrahepatic vascular resistance and pressure regulation [54]. Furthermore, animal experiments have shown that HSC activation is often accompanied by an increase in the level of TGF-*β*1 and that inhibition of TGF-*β*1 synthesis, as well as TGF-*β* receptor blockade, can significantly reduce the pressure of portal hypertension [55]. Therefore, intervention of this signaling pathway (particularly inhibition of TGF-*β*1 levels) may be an important target for the prevention and treatment of portal hypertension due to liver cirrhosis.

miRNAs have been reported to be central players in antifibrotic and profibrotic signaling pathways and in related gene regulation during HSC myofibroblastic transdifferentiation [51]. HSC activation is a pivotal event in the initiation and progression of hepatic fibrosis and a major contributor to collagen deposition driven by TGF-*β*. Inhibition of TGF-*β* signaling by miR-19b was confirmed by decreased expression of TGF-*β* signaling components, such as TGF-*β* receptor II (TGF-*β*RII) and SMAD3, which further blocked TGF-*β*-induced expression of *α*1(I) and *α*2(I) procollagen and COL1A1 mRNAs [56]. Recently, He et al. [57] reported that HSC miR-146a expression was downregulated in a dose-dependent manner in response to TGF-*β*1 stimulation, as observed with one-step real-time quantitative RT-PCR. Moreover, He and colleagues confirmed that the overexpression of miR-146a suppressed TGF-*β*-induced HSC proliferation and increased the HSC apoptosis index by targeting SMAD4. As for the TGF-*β*/SMAD signaling pathway, SMAD3 is considered to function as an R-smad and SMAD4 functioned as Co-SMAD, while SMAD7 functioned as an anti-SMAD. Elevated miR-21 has been observed to act as a profibrogenic miRNA by its repression of the TGF-*β* inhibitory SMAD-7 protein [58].

Although some effects of a variety of signaling pathways in reducing intrahepatic vascular resistance are important, they are not sufficient to block intrahepatic portal hypertension. Recent data suggest that intrahepatic angiogenesis may also be involved in sinusoidal systemic circulation and portal hypertension [11]. In cirrhosis, tissue hypoxia is postulated to stimulate angiogenesis [59]. Hypoxia stimulates the production of VEGF, which is one of the most important angiogenic growth factors [60]. Recent studies also suggest that HSCs are the major contributors to angiogenesis [61]. This may occur through direct and indirect mechanisms. The former include the activation and proliferation of HSCs, which require tightly regulated autocrine and/or paracrine fibrogenetic factors such as VEGF [62]. Conversely, indirect mechanisms are also likely to be important and include the ability of HSCs to secrete angiogenic factors that promote angiogenesis [63]. With the initiation of angiogenesis, the collateral circulation in the liver is formed. This increases sinusoidal blood flow, leading to increased portal pressure [11]. Experiments have shown that treatment of portal hypertensive rats with SU5416 (a specific inhibitor of the VEGF receptor) resulted in a significant and marked decrease (44%) in portal venous inflow and decreased portal venous resistance by 93% [64].

**Figure 2:**
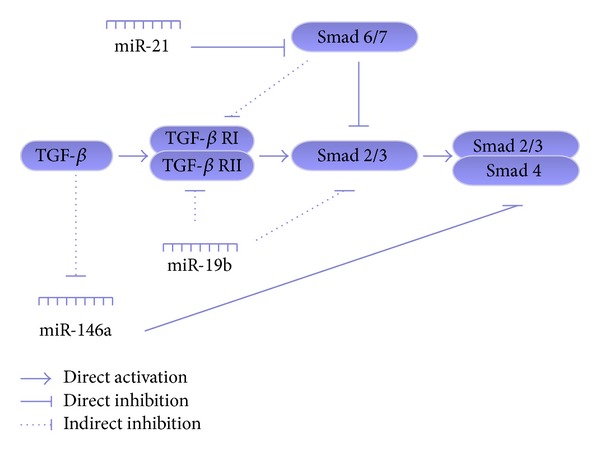
Summary of the miRNAs and the TGF-*β* pathway involved in HSCs. The particular TGF-*β* signaling pathway affected by miRNAs is described in detail in the review.

**Figure 3:**
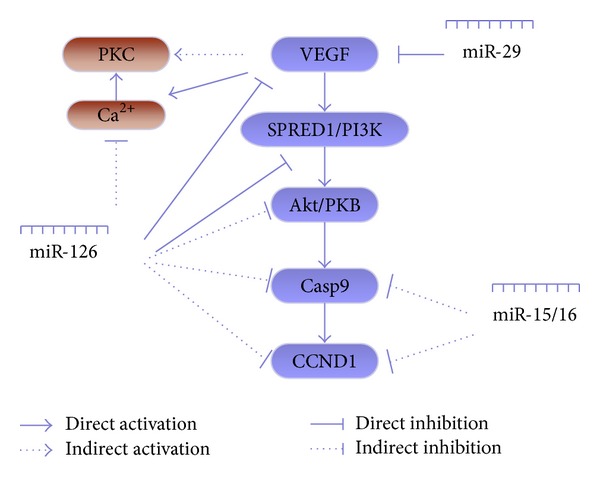
Summary of the miRNAs and the VEGF pathway involved in HSCs. The particular VEGF signaling pathway affected by miRNAs is described in detail in the review.

Therefore, small-molecule inhibitors that target the growth factor pathways leading to angiogenesis and vascular resistance (i.e., VEGF, TGF-*β*1) can lower portal hypertension. This is probably through dual and converging antifibrogenic and antiangiogenic actions that affect HSCs. Fortunately, miRNAs have emerged as new players of gene regulation during angiogenesis in vascular diseases [65]. The VEGF family and related pathways are widely known as the most potent angiogenic inducers during angiogenesis, vasculogenesis, and tumorigenesis [66]. Plenty of miRNAs have been associated with these pro- and antiangiogenic factors. For example, miR-29 is downregulated during fibrosis and acts as an antifibrogenic mediator not only by targeting collagen biosynthesis but also by interfering with proangiogenic factors via PDGF-C, VEGF-A, and IGF-I [43]. Aquaporin-1 (AQP1), confirmed to be regulated by osmotically sensitive miRNAs, promotes angiogenesis, fibrosis, and portal hypertension after bile duct ligation [67]. The miR-126 family is considered to be associated with angiogenesis and is implicated in vessel development and vascular integrity by directly repressing negative regulators of the VEGF pathway, including the Sprouty-related protein SPRED1 and phosphoinositol-3 kinase regulatory subunit 2 (PIK3R2/p85-beta) [42]. Recent studies confirmed that restoring HSCs with Lv-miR-126* resulted in decreased proliferation, accumulation of ECM components, and cell contraction, while also negatively regulating the VEGF/PI3k/Akt/CCND1 pathway and VEGF-A/Ca^2+^ pathway by partially targeting VEGF-A [68]. These findings illustrate that a single miRNA can regulate vascular integrity and angiogenesis, providing a new target for modulating intrahepatic microcirculation (Figures 2 and 3).

Apart from TGF-*β*-regulated fibrogenesis and VEGF-induced angiogenesis, other signaling pathways relevant to the biological behavior of HSCs in intrahepatic portal hypertension have been reported previously in rat HSCs during activation [69]. Nevertheless, the use of microRNA therapies remains uncertain and is poorly applied in clinical portal hypertension. Increasing the knowledge and the translational potential of this idea is critical for its realization.

## 5. Therapeutic Delivery of miRNAs to the Liver

In vivo delivery of miRNAs is central to miRNA-based therapy. Different methods, using mainly viral and nonviral vehicles, have been developed to increase the targeting and efficiency of miRNAs. For nonviral vehicles, a liposomal delivery system, YSK05-MEND, was used to systemically administer anti-miRNA oligonucleotides (AMOs) to the livers of mice at a low dose [70]. A cationic lipid-based nanoparticle system reflects a novel systemic delivery agent for miRNA. Intravenous administration of interfering nanoparticles (iNOPs), which are prepared by lipid-functionalized poly-L-lysine dendrimer, results in 83% specific silencing of target miRNA. The specific silencing of miR-122 by iNOP-7 is long-lasting and does not induce an immune response [71]. Lipid-based nanoparticles (LNPs) containing oleic acid (OA), an unsaturated fatty acid, also demonstrate the delivery efficacy of miRNA and the inhibition of the target (Bcl-w) to a greater degree than with Lipofectamine 2000 [72]. A peptide vector has recently proved to be another type of miRNA delivery system. MPG, which is a 27-residue peptide vector containing the hydrophobic domain derived from the fusion sequence of HIV-1 gp41 and the hydrophilic domain derived from the nuclear localization sequence of SV40 T-antigen, is capable of delivering both miR-122 mimic and inhibitor into mouse liver cells and effectively regulating cholesterol levels [73]. On the other side, viral vectors, including adenovirus, adeno-associated virus (AAV), and lentivirus, have been widely employed to deliver miRNAs into liver cells (hepatocytes, HSCs, etc.).

## 6. Conclusion and Perspective

In contrast to traditional inhibitors and monoclonal antibodies that target one specific protein, miRNAs exhibit a unique, namely, multitargeted, pattern of action. Limited complementarity between “seed sequence” of single miRNA and 3′ untranslated region (3′UTR) of multiple genes, most of which are recognized to be the members of signal pathways, is sufficient to downregulate their mRNA and/or protein levels [74, 75]. As a result, different signal pathways may be under the control of one miRNA.

A lot of signal pathways (TGF-*β*, VEGF, Apoptosis, etc.), concerning different phenotypes (apoptosis, collagen production and secretion, etc.), are involved in the biological behavior of HSCs. Most of them may serve as the therapeutic targets of liver fibrosis. Notably, TGF-*β* and VEGF are two important signaling pathways that may promote portal hypertension through multiple mechanisms [68, 76]. No validated treatments exist for these two targets simultaneously; however, miRNA therapy offers novel possibilities. Hence, restoring some intracellular miRNAs may lead to reduced HSC proliferation and contractility and suppressed angiogenesis by targeting the TGF-*β* and VEGF-mediated signaling pathway, thereby reducing hepatic portal shunting and improving the sinusoidal microcirculation. These findings may not only increase our current knowledge about the significance of HSC biological behavior, but may also provide a novel therapeutic strategy against intrahepatic vascular resistance and increased portal blood flow in chronic liver diseases.

## Figures and Tables

**Table 1:** MicroRNAs linked to HSCs.

miRNA	Predicted target and confirmation level	Putative pathway in fibrosis	References	Expression during HSCs activation
miR-15b	Reporter gene assay (Bcl-2)	Apoptosis	[26]	↓
miR-16	Reporter gene assay (Bcl-2) Target protein changes (CCND1)	Apoptosis cell cycle and cell proliferation	[26, 29]	↓
miR-126	Reporter gene assay (VEGFA)	VEGF/PI3k/Akt/CCND1 pathway and VEGF-A/Ca^2+^ pathway	[42]	↓
miR-122	Target mRNA changes (P4HA1)	Collagen production	[27]	↓
miR-150	Target protein changes (cmyb)	Cell activation and proliferation	[23]	↓
miR-19b	Reporter gene assays (TGF-*β*RII)	TGF-*β* signaling	[37]	↓
miR-194	Target protein changes (rac1)	Cell activation and proliferation	[32]	↓
miR-195	Reporter gene assays (cyclin E1)	Cell proliferation	[35]	↓
miR-29 family	Reporter gene assays (different collagens, PDGF-C, VEGF-A, and IGF-I)	ECM synthesis	[34, 40, 43]	↓
miR-335	Target mRNA changes (tenascin-C)	Cell activation and migration	[25]	↓
miR-146a	Reporter gene assays (smad4)	TGF-*β* signaling	[33]	↓
miR-34a	Reporter gene assay (ACSL1)	Lipids biosynthesis	[28]	↑
miR-27b, miR-27a	Reporter gene assay (RXR-a)	Lipid accumulation and cell proliferation	[30]	↑
miR-199a/b	Reporter gene assay (Dyrk1)	ECM synthesis via calcineurin/NFAT signaling	[28]	↑
miR-221/222	Reporter gene assay (CDKN1B)	Cell activation	[24]	↑
miR-21	Target protein changes (PTEN)	PTEN/Akt pathway	[31]	↑
miR-181b	Reporter gene assay (P27)	Cell proliferation	[36]	↑

## References

[1] Leuci D, Pomarico G, Casucci N, Fucci B, D’Alitto N, Caldarone A (2008). Primary prophylaxis of esophageal variceal bleeding in cirrhotic patients. *Recenti Progressi in Medicina*.

[2] Jiao LR, Inglott FS, Mathie RT, Habib NA (2001). The effect of augmenting portal venous inflow on intrahepatic pressure and resistance in the isolated perfused porcine liver. *Hepato-Gastroenterology*.

[3] Reynaert H, Thompson MG, Thomas T, Geerts A (2002). Hepatic stellate cells: role in microcirculation and pathophysiology of portal hypertension. *Gut*.

[4] Kitao A, Sato Y, Sawada-Kitamura S (2009). Endothelial to mesenchymal transition via transforming growth factor-*β*1/Smad activation is associated with portal venous stenosis in idiopathic portal hypertension. *The American Journal of Pathology*.

[5] Fernandez M, Mejias M, Garcia-Pras E, Mendez R, Garcia-Pagan JC, Bosch J (2007). Reversal of portal hypertension and hyperdynamic splanchnic circulation by combined vascular endothelial growth factor and platelet-derived growth factor blockade in rats. *Hepatology*.

[6] Shah AA, Leidinger P, Blin N, Meese E (2010). miRNA: small molecules as potential novel biomarkers in cancer. *Current Medicinal Chemistry*.

[7] Xiong L, Jiang W, Zhou R, Mao C, Guo Z (2013). Identification and analysis of the regulatory network of Myc and microRNAs from high-throughput experimental data. *Computers in Biology and Medicine*.

[8] Ambros V (2004). The functions of animal microRNAs. *Nature*.

[9] Liu Y, Chen SH, Jin X, Li YM (2013). Analysis of differentially expressed genes and microRNAs in alcoholic liver disease. *International Journal of Molecular Medicine*.

[10] Hernandez-Gea V, Turon F, Berzigotti A, Villanueva A (2013). Management of small hepatocellular carcinoma in cirrhosis: focus on portal hypertension. *World Journal of Gastroenterology*.

[11] Thabut D, Shah V (2010). Intrahepatic angiogenesis and sinusoidal remodeling in chronic liver disease: new targets for the treatment of portal hypertension?. *Journal of Hepatology*.

[12] Thimgan MS, Yee HF (1999). Quantitation of rat hepatic stellate cell contraction: stellate cells’ contribution to sinusoidal resistance. *American Journal of Physiology: Gastrointestinal and Liver Physiology*.

[13] Friedman SL (2004). Mechanisms of disease: mechanisms of hepatic fibrosis and therapeutic implications. *Nature Clinical Practice Gastroenterology and Hepatology*.

[14] Fallowfield A Hayden J, Snowdon V (2013). Relaxin modulates human and rat hepatic myofibroblast function and ameliorates portal hypertension *in vivo*. *Hepatology*.

[15] Pinzani M, Marra F (2001). Cytokine receptors and signaling in hepatic stellate cells. *Seminars in Liver Disease*.

[16] Gupta TK, Chen L, Groszmann RJ (1997). Pathophysiology of portal hypertension. *Baillière's Clinical Gastroenterology*.

[17] Reynaert H, Urbain D, Geerts A (2008). Regulation of sinusoidal perfusion in portal hypertension. *Anatomical Record*.

[18] Lee JS, Kim JH (2007). The role of activated hepatic stellate cells in liver fibrosis, portal hypertension and cancer angiogenesis. *The Korean Journal of Hepatology*.

[19] Fernandez M, Mejias M, Garcia-Pras E, Mendez R, Garcia-Pagan JC, Bosch J (2007). Reversal of portal hypertension and hyperdynamic splanchnic circulation by combined vascular endothelial growth factor and platelet-derived growth factor blockade in rats. *Hepatology*.

[20] Kim MY, Baik SK (2010). Pathophysiology of portal hypertension, what’s new?. *The Korean Journal of Gastroenterology*.

[21] Jiang F, Parsons CJ, Stefanovic B (2006). Gene expression profile of quiescent and activated rat hepatic stellate cells implicates Wnt signaling pathway in activation. *Journal of Hepatology*.

[22] Maubach G, Lim MC, Chen J, Yang H, Zhuo L (2011). miRNA studies in *in vitro* and *in vivo* activated hepatic stellate cells. *World Journal of Gastroenterology*.

[23] Zheng J, Lin Z, Dong P (2013). Activation of hepatic stellate cells is suppressed by microRNA-150. *International Journal of Molecular Medicine*.

[24] Ogawa T, Enomoto M, Fujii H (2012). MicroRNA-221/222 upregulation indicates the activation of stellate cells and the progression of liver fibrosis. *Gut*.

[25] Chen C, Wu C-Q, Zhang Z-Q, Yao D-K, Zhu L (2011). Loss of expression of miR-335 is implicated in hepatic stellate cell migration and activation. *Experimental Cell Research*.

[26] Guo C-J, Pan Q, Li D-G, Sun H, Liu B-W (2009). miR-15b and miR-16 are implicated in activation of the rat hepatic stellate cell: an essential role for apoptosis. *Journal of Hepatology*.

[27] He Y, Huang C, Zhang SP, Sun X, Long X-R, Li J (2012). The potential of microRNAs in liver fibrosis. *Cellular Signalling*.

[28] Vettori S, Gay S, Distler O (2012). Role of microRNAs in fibrosis. *The Open Rheumatology Journal*.

[29] Guo C-J, Pan Q, Jiang B, Chen G-Y, Li D-G (2009). Effects of upregulated expression of microRNA-16 on biological properties of culture-activated hepatic stellate cells. *Apoptosis*.

[30] Ji J, Zhang J, Huang G, Qian J, Wang X, Mei S (2009). Over-expressed microRNA-27a and 27b influence fat accumulation and cell proliferation during rat hepatic stellate cell activation. *FEBS Letters*.

[31] Wei J, Feng L, Li Z, Xu G, Fan X (2013). MicroRNA-21 activates hepatic stellate cells via PTEN/Akt signaling. *Biomedicine & Pharmacotherapy*.

[32] Meng Z, Fu X, Chen X (2010). miR-194 is a marker of hepatic epithelial cells and suppresses metastasis of liver cancer cells in mice. *Hepatology*.

[33] He Y, Huang C, Sun X, Long X-R, Lv X-W, Li J (2012). MicroRNA-146a modulates TGF-beta1-induced hepatic stellate cell proliferation by targeting SMAD4. *Cellular Signalling*.

[34] Sekiya Y, Ogawa T, Yoshizato K, Ikeda K, Kawada N (2011). Suppression of hepatic stellate cell activation by microRNA-29b. *Biochemical and Biophysical Research Communications*.

[35] Sekiya Y, Ogawa T, Iizuka M, Yoshizato K, Ikeda K, Kawada N (2011). Down-regulation of cyclin E1 expression by microRNA-195 accounts for interferon-*β*-induced inhibition of hepatic stellate cell proliferation. *Journal of Cellular Physiology*.

[36] Wang B, Li W, Guo K, Xiao Y, Wang Y, Fan J (2012). miR-181b Promotes hepatic stellate cells proliferation by targeting p27 and is elevated in the serum of cirrhosis patients. *Biochemical and Biophysical Research Communications*.

[37] Steuerwald NM, Walling TL, Ghosh S (2012). Inhibitory effects of microRNA 19b in hepatic stellate cell-mediated fibrogenesis. *Hepatology*.

[38] Thompson KJ, Mckillop IH, Schrum LW (2011). Targeting collagen expression in alcoholic liver disease. *World Journal of Gastroenterology*.

[39] McCuskey RS (2008). The hepatic microvascular system in health and its response to toxicants. *Anatomical Record*.

[40] Ogawa T, Iizuka M, Sekiya Y, Yoshizato K, Ikeda K, Kawada N (2010). Suppression of type I collagen production by microRNA-29b in cultured human stellate cells. *Biochemical and Biophysical Research Communications*.

[41] Yang Y-Y, Tsai T-H, Huang Y-T (2012). Hepatic endothelin-1 and endocannabinoids-dependent effects of hyperleptinemia in nonalcoholic steatohepatitis-cirrhotic rats. *Hepatology*.

[42] Fish JE, Santoro MM, Morton SU (2008). miR-126 regulates angiogenic signaling and vascular integrity. *Developmental Cell*.

[43] Zhu H, Fan GC (2012). Role of microRNAs in the reperfused myocardium towards post-infarct remodelling. *Cardiovascular Research*.

[44] Noetel A, Elfimova N, Altmüller J (2013). Next generation sequencing of the Ago2 interacting transcriptome identified chemokine family members as novel targets of neuronal microRNAs in hepatic stellate cells. *Journal of Hepatology*.

[45] Yu F, Lin Z, Zheng J, Gao S, Lu Z, Dong P (2014). Suppression of collagen synthesis by Dicer gene silencing in hepatic stellate cells. *Molecular Medicine Reports*.

[46] Liu Y, Wen XM, Lui ELH (2009). Therapeutic targeting of the PDGF and TGF-*Β*-signaling pathways in hepatic stellate cells by PTK787/ZK22258. *Laboratory Investigation*.

[47] Sauvant P, Cansell M, Atgie C (2011). Vitamin A and lipid metabolism: relationship between hepatic stellate cells (HSCs) and adipocytes. *Journal of Physiology and Biochemistry*.

[48] Kong D, Zhang F, Wei D (2013). Paeonol inhibits hepatic fibrogenesis via disrupting nuclear factor-*κ*B pathway in activated stellate cells: *in vivo* and *in vitro* studies. *Journal of Gastroenterology and Hepatology*.

[49] Zhu N-L, Asahina K, Wang J (2012). Hepatic stellate cell-derived delta-like homolog 1 (DLK1) protein in liver regeneration. *The Journal of Biological Chemistry*.

[50] Zhao Y, Wang Y, Wang Q, Liu Z, Liu Q, Deng X (2012). Hepatic stellate cells produce vascular endothelial growth factor via phospho-p44/42 mitogen–activated protein kinase/cyclooxygenase-2 pathway. *Molecular and Cellular Biochemistry*.

[51] Guo C-J, Pan Q, Cheng T, Jiang B, Chen G-Y, Li D-G (2009). Changes in microRNAs associated with hepatic stellate cell activation status identify signaling pathways. *FEBS Journal*.

[52] Cao S, Yaqoob U, Das A (2010). Neuropilin-1 promotes cirrhosis of the rodent and human liver by enhancing PDGF/TGF-*β* signaling in hepatic stellate cells. *The Journal of Clinical Investigation*.

[53] Majumder S, Piguet AC, Dufour JF, Chatterjee S (2013). Study of the cellular mechanism of sunitinib mediated inactivation of activated hepatic stellate cells and its implications in angiogenesis. *European Journal of Pharmacology*.

[54] Breitkopf K, Godoy P, Ciuclan L, Singer MV, Dooley S (2006). TGF-*β*/Smad signaling in the injured liver. *Zeitschrift für Gastroenterologie*.

[55] Liang J, Deng X, Lin Z-X, Zhao L-C, Zhang X-L (2009). Attenuation of portal hypertension by natural taurine in rats with liver cirrhosis. *World Journal of Gastroenterology*.

[56] Lakner AM, Steuerwald NM, Walling TL (2012). Inhibitory effects of microRNA 19b in hepatic stellate cell-mediated fibrogenesis. *Hepatology*.

[57] He Y, Huang C, Sun X, Lv X-W, Li J (2012). MicroRNA-146a modulates TGF-beta1-induced hepatic stellate cell proliferation by targeting SMAD4. *Cellular Signalling*.

[58] Noetel A, Kwiecinski M, Elfimova N, Huang J, Odenthal M (2012). MicroRNA are central players in anti- and profibrotic gene regulation during liver fibrosis. *Frontiers in Physiology*.

[59] Rosmorduc O, Housset C (2010). Hypoxia: a link between fibrogenesis, angiogenesis, and carcinogenesis in liver disease. *Seminars in Liver Disease*.

[60] Corpechot C, Barbu V, Wendum D (2002). Hypoxia-induced VEGF and collagen I expressions are associated with angiogenesis and fibrogenesis in experimental cirrhosis. *Hepatology*.

[61] Zhao Y, Wang Y, Wang Q, Liu Z, Liu Q, Deng X (2012). Hepatic stellate cells produce vascular endothelial growth factor via phospho-p44/42 mitogen-activated protein kinase/cyclooxygenase-2 pathway. *Molecular and Cellular Biochemistry*.

[62] Valfre di Bonzo L, Novo E, Cannito S (2009). Angiogenesis and liver fibrogenesis. *Histology and Histopathology*.

[63] Taura K, Minicis S, Seki E (2008). Hepatic stellate cells secrete angiopoietin 1 that induces angiogenesis in liver fibrosis. *Gastroenterology*.

[64] Fernandez M, Mejias M, Angermayr B, Garcia-Pagan JC, Rodes J, Bosch J (2005). Inhibition of VEGF receptor-2 decreases the development of hyperdynamic splanchnic circulation and portal-systemic collateral vessels in portal hypertensive rats. *Journal of Hepatology*.

[65] Urbich C, Kuehbacher A, Dimmeler S (2008). Role of microRNAs in vascular diseases, inflammation, and angiogenesis. *Cardiovascular Research*.

[66] Zhou Y, McMaster M, Woo K (2002). Vascular endothelial growth factor ligands and receptors that regulate human cytotrophoblast survival are dysregulated in severe preeclampsia and hemolysis, elevated liver enzymes, and low platelets syndrome. *The American Journal of Pathology*.

[67] Huebert RC, Jagavelu K, Hendrickson HI (2011). Aquaporin-1 promotes angiogenesis, fibrosis, and portal hypertension through mechanisms dependent on osmotically sensitive microRNAs. *The American Journal of Pathology*.

[68] Guo CJ, Pan Q, Xiong H (2013). Dynamic expression of miR-126 and its effects on proliferation and contraction of hepatic stellate cells. *FEBS Letters*.

[69] Thabut D, Routray C, Lomberk G (2011). Complementary vascular and matrix regulatory pathways underlie the beneficial mechanism of action of sorafenib in liver fibrosis. *Hepatology*.

[70] Takahashi M, Yamada N, Hatakeyama H (2013). *In vitro* optimization of 2′-OMe-4′-thioribonucleoside-modified anti-microRNA oligonucleotides and its targeting delivery to mouse liver using a liposomal nanoparticle. *Nucleic Acids Research*.

[71] Baigude H, Rana TM (2012). Interfering nanoparticles for silencing microRNAs. *Methods in Enzymology*.

[72] Wang X, Yu B, Ren W (2013). Enhanced hepatic delivery of siRNA and microRNA using oleic acid based lipid nanoparticle formulations. *Journal of Controlled Release*.

[73] Wang L, Tang W, Yan S (2013). Efficient delivery of miR-122 to regulate cholesterol metabolism using a non-covalent peptide-based strategy. *Molecular Medicine Reports*.

[74] Zhang Y, Fan KJ, Sun Q (2012). Functional screening for miRNAs targeting Smad4 identified miR-199a as a negative regulator of TGF-*β* signalling pathway. *Nucleic Acids Research*.

[75] Zhang Y, Wang Z, Gemeinhart RA (2013). Progress in microRNA delivery. *Journal of Controlled Release*.

[76] Hidaka H, Nakazawa T, Shibuya A (2011). Effects of 1-year administration of olmesartan on portal pressure and TGF-beta1 in selected patients with cirrhosis: a randomized controlled trial. *Journal of Gastroenterology*.

